# Finite element analysis of Tumor Treating Fields in a patient with posterior fossa glioblastoma

**DOI:** 10.1007/s11060-020-03406-x

**Published:** 2020-01-27

**Authors:** Edwin Lok, Pyay San, Olivia Liang, Victoria White, Eric T. Wong

**Affiliations:** Brain Tumor Center & Neuro-Oncology Unit, Harvard Medical School, Beth Israel Deaconess Medical Center, 330 Brookline Avenue, Boston, MA 02215 USA

**Keywords:** Glioblastoma, Posterior fossa, Tumor treating fields, Modeling, Finite element analysis

## Abstract

**Introduction:**

Tumor Treating Fields (TTFields) are alternating electric fields at 200 kHz that disrupt tumor cells as they undergo mitosis. Patient survival benefit has been demonstrated in randomized clinical trials but much of the data are available only for supratentorial glioblastomas. We investigated a series of alternative array configurations for the posterior fossa to determine the electric field coverage of a cerebellar glioblastoma.

**Methods:**

Semi-automated segmentation of neuro-anatomical structures was performed while the gross tumor volume (GTV) was manually delineated. A three-dimensional finite-element mesh was generated and then solved for field distribution.

**Results:**

Compared to the supratentorial array configuration, the alternative array configurations consist of posterior displacement the 2 lateral opposing arrays and inferior displacement of the posteroanterior array, resulting in an average increase of 46.6% electric field coverage of the GTV as measured by the area under the curve of the electric field-volume histogram (E_AUC_). Hotspots, or regions of interest with the highest 5% of TTFields intensity (E_5%_), had an average increase of 95.6%. Of the 6 posterior fossa configurations modeled, the PA_Horizontal_ arrangement provided the greatest field coverage at the GTV when the posteroanterior array was placed centrally along the patient’s posterior neck and horizontally parallel, along the longer axis, to the coronal plane of the patient’s head. Varying the arrays also produced hotspots proportional to TTFields coverage.

**Conclusions:**

Our finite element modeling showed that the alternative array configurations offer an improved TTFields coverage to the cerebellar tumor compared to the conventional supratentorial configuration.

## Introduction

Tumor Treating Fields (TTFields) therapy is an accepted treatment modality for supratentorial glioblastoma because of its ability to prolong the survival of patients. TTFields are delivered to the scalp via 2 pairs of orthogonally positioned transducer arrays and, at a frequency of 200 kHz, these alternating electric fields can penetrate the scalp and calvarium into the intracranial space [1], producing an antitumor effect by disrupting tumor cell cytokinesis during mitosis and enabling immunogenic cell death [2] When applied to newly diagnosed glioblastoma patients in a randomized clinical trial, those who received TTFields and adjuvant temozolomide had an improved overall survival of 20.9 months compared to the 16.0 months in the control cohort treated with adjuvant temozolomide alone [3]. The median progression-free survival was also prolonged to 6.7 months from 4.0 months [3]. The only unique adverse event related to TTFields is the mild to moderate scalp irritation at the sites of array application [4]. Collectively, these data indicate that TTFields offer substantial clinical benefit to glioblastoma patients with acceptable toxicity.

The standard array placement configuration is designed for the delivery of TTFields to tumors located in the supratentorial brain [5, 6]. But there is substantial variability among patients with respect to the intracranial distribution of TTFields and, depending on the array layouts, the electric field coverage at the gross tumor volume (GTV) can vary up to 23% according to a computer simulation study [7]. For tumors in the posterior fossa, the standard array configuration appears to provide minimal electric field coverage [8] and there is no accepted array placement for this location. Therefore, we performed a finite element computer simulation study of a patient with a cerebellar glioblastoma who is also undergoing TTFields treatment using a modified array configuration. In this configuration, the posteroanterior (PA) array is shifted downward to the lower occipital and upper cervical regions, and the right and left lateral arrays are moved backward. Our simulation demonstrated that this posterior fossa configuration significantly increased the electric field coverage to the cerebellar GTV, as measured by the area under the curve (AUC) of the electric field-volume histogram (EVH). In addition, the modified array configuration produces hotspots proportional to TTFields coverage at the cerebellar GTV.

## Methods

MP RAGE, T1 and T2 MRI sequences from a 63-year-old woman with a glioblastoma located in the posterior fossa were used to perform finite element analysis according to an IRB protocol approved by Dana Farber Cancer Institute. Semi-automated segmentation of neuro-anatomical structures was performed using methods previously described [9]. Briefly, various intracranial structures from the automated segmentation were imported into Simpleware (Exeter, UK) where unspecified and unsegmented structures, such as muscles, blood vessels, parotid glands, vertebral body, mandible, tongue, epidural tissues, GTV and necrotic core(s) within the GTV were delineated.

**Fig. 1:**
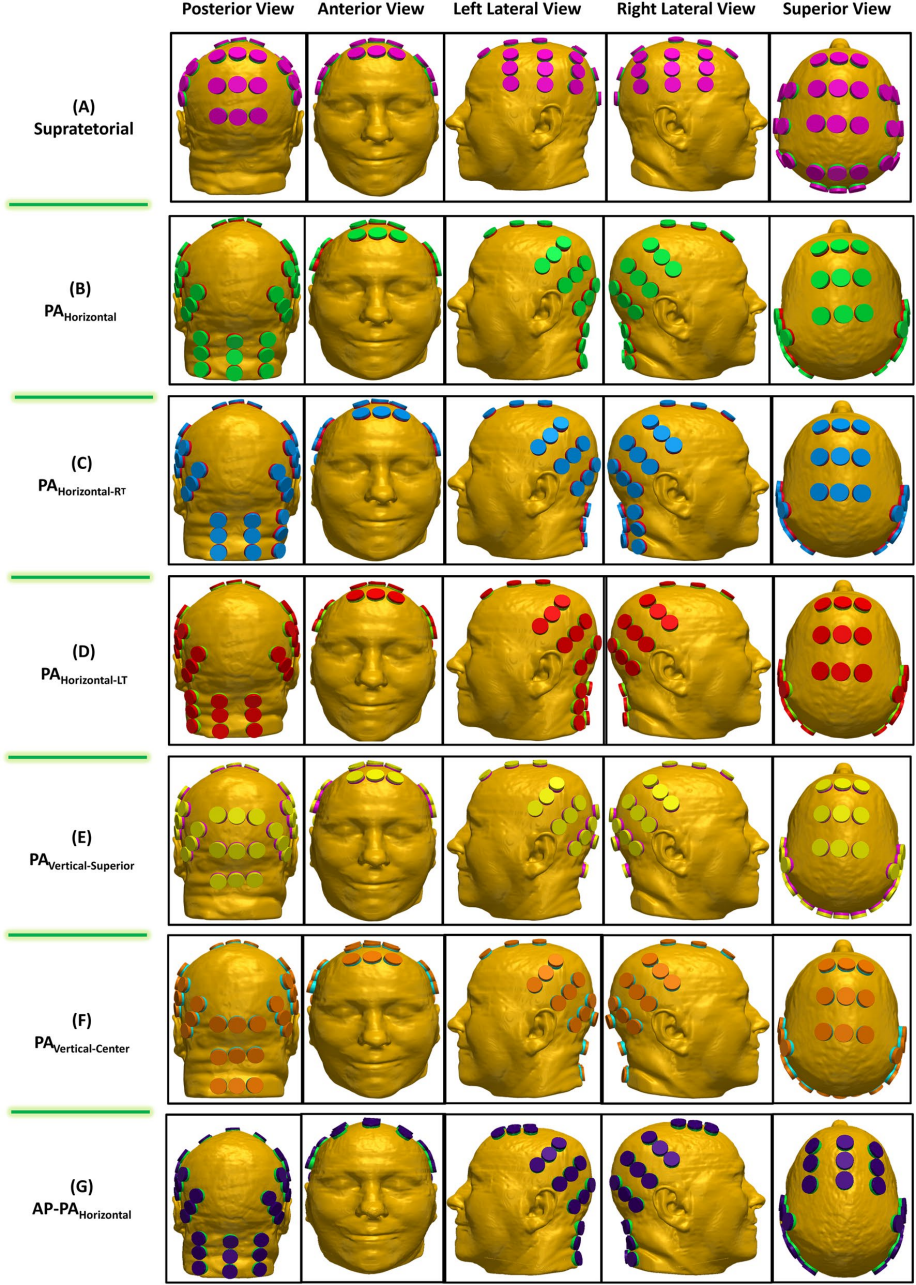
Various array configuration layouts. The supratentorial configuration was determined by the NovoTAL™ software that generated the array layout (**a**). Variations of the alternative array configurations by rotating the PA array 90° where the array is parallel to the coronal plane with the longer axis running from left to right and shifting it laterally to the left and right (**b**–**d**). Additionally, 2 other alternative array configurations were applied by where the PA array’s longer axis runs superior to inferior but parallel to the coronal plane and shifted superiorly (**e**, **f**). **g** shows the same configuration as (**b**) except the AP array is rotated by 90°

**Fig. 2:**
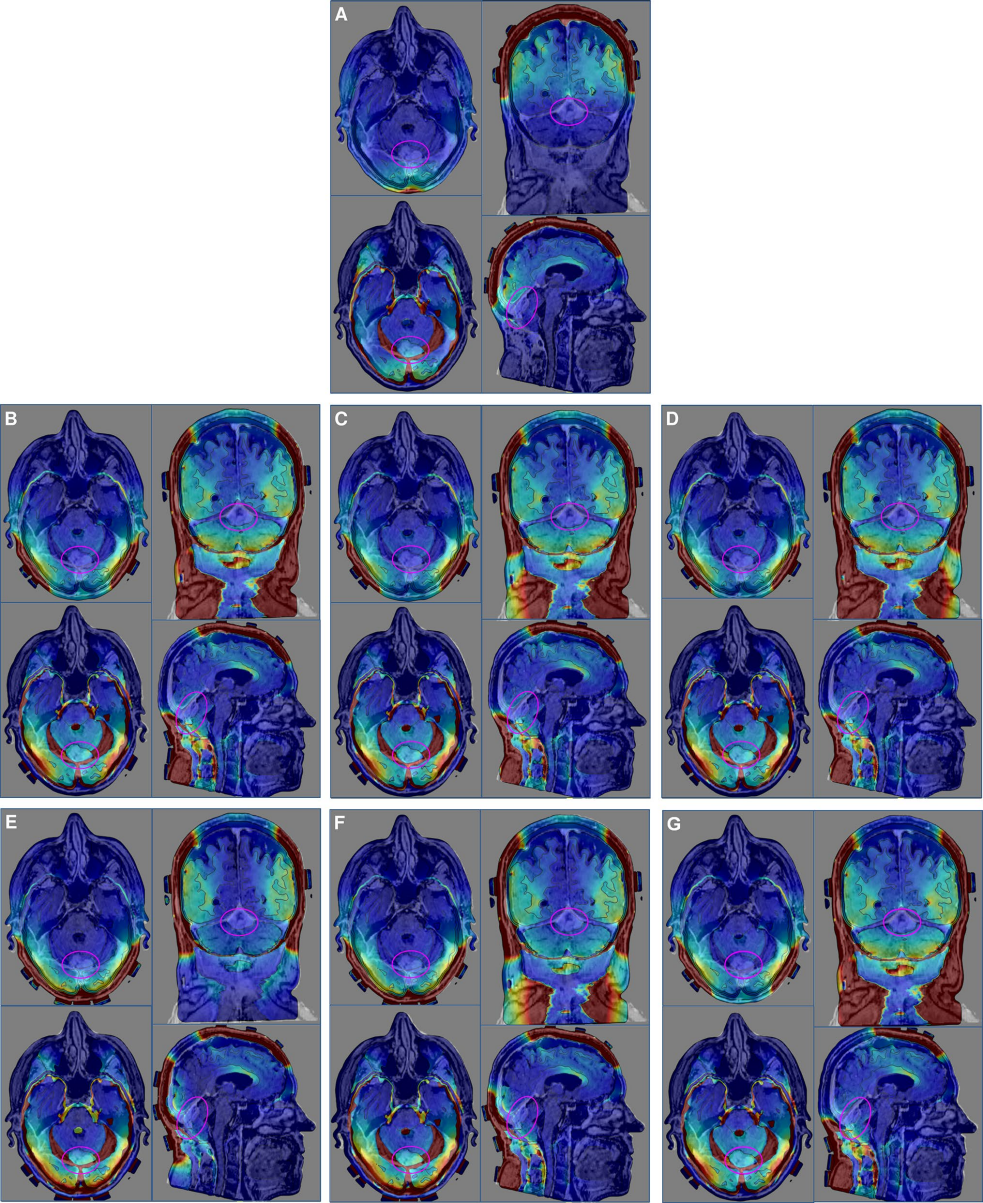
Electric field and current density distribution overlays. In each panel, upper left corner is an axial plane with electric field overlay, while the lower left corner is an axial plane with current density overlay. The upper right corner in each panel is a coronal plane with the electric field overlay, while the lower right corner is a sagittal plane with the electric field overlay. **a** Supratentorial array configuration. **b** Alternative array configuration with PA_Horizontal_. **c** Alternative array configuration with PA_Horizontal-RT_. **d** Alternative array configuration with PA_Horizontal-LT_. **e** Alternative array configuration with PA_Vertical-Superior_. **f** Alternative array configuration with PA_Vertical-Center_. **g** Alternative array configuration with AP-PA_Horizontal_

The distribution of TTFields was investigated using two major transducer array configurations: (i) the conventional supratentorial array configuration (Fig. 1a) generated by NovoTAL (a proprietary treatment planning software from Novocure, LTD.) and (ii) the alternative array configurations developed for tumors within the posterior fossa. In one of the posterior fossa array configurations, the two lateral and the PA arrays were shifted from the supratentorial array configuration and this is denoted as PA_Vertical-Center_ (Fig. 1f). The PA array was then manually shifted from the PA_Vertical-Center_ position to 5 different positions and they were labeled as PA_Horizontal_ (Fig. 1b), PA_Horizontal-Right_ (Fig. 1c), PA_Horizontal-Left_ (Fig. 1d), PA_Vertical-Superior_ (Fig. 1e), and AP-PA_Horizontal_ (Fig. 1g). A three-dimensional finite-element mesh was then generated after the segmentation was completed and reviewed by a neuro-oncologist for accuracy. The mesh was then imported into COMSOL Multiphysics (Burlington, MA) for finite element analysis. MRI overlays of electric field and current density distributions were constructed corresponding to the conventional supratentorial array configuration (Fig. 2a) and various alternative array configurations, including PA_Horizontal_ (Fig. 2b), PA_Horizontal-Right_ (Fig. 2c), PA_Horizontal-Left_ (Fig. 2d), PA_Vertical-Superior_ (Fig. 2e), PA_Vertical-Center_ (Fig. 2f) and AP-PA_Horizontal_ (Fig. 2g).

To compare TTFields coverage and intensity between models, electric field-volume histogram (EVH) and current density volume histogram (CDVH) were first generated. Plan Quality Metrics (PQM) were then derived from these histograms for the purpose of quantitative comparisons. The overall field coverage of the GTV and the cerebellum among various array placements were also compared using the metrics of the area under the curve in the EVH (E_AUC_) and in the CDVH (CD_AUC_) for electric field and current density, respectively. The median volume for TTFields covering the GTV and cerebellum were also compared between the different array configurations and they are denoted as E_50%_ and CD_50%_. In order to compare field intensities much higher than what was received by 50% in the region of interest (ROI), we defined these hotspot regions as 5% of the total volume of the ROI and denoted them as E_5%_ and CD_5%_.

## Results

### Array positioning greatly affects TTFields coverage of the GTV in the posterior fossa

The posterior fossa configurations consist of posterior displacement of the 2 lateral opposing arrays and the PA array, resulting in an average increase of 48.0% in E_AUC_ and 41.9% in CD_AUC_ at the GTV when compared to the supratentorial array configuration (Table 1). Since the GTV was located dorsally at the midline, and the patient is routinely required to shift the arrays by 2 cm laterally during array exchange in order to reduce the amount of scalp erythema, we asked whether or not lateral and superior shifts, as well as rotations of the PA array (which was closest to the GTV), would have a profound effect on TTFields coverage at the GTV.

**Table 1:** PQM of electric field coverage and current density for various array configurations within the GTV and the cerebellum

ROI	Array configuration	Electric fields PQM						
		E_AUC_	V_E75_	V_E50_	V_E25_	E_75%_	E_50%_	E_5%_
	Units	(V/m)	(%)			(V/m)		
GTV	(A) Supratentorial	26.4	0.1	1.9	64.6	22.6	28.0	44.1
	(B) PA horizontal	41.0	10.4	28.2	71.6	22.8	38.1	99.1
	(C) PA horiz RT	39.2	9.9	24.0	69.5	21.7	36.4	95.6
	(D) PA horiz LT	39.9	9.9	25.5	71.0	22.5	37.0	95.7
	(E) PA VRT SUP	34.4	1.2	18.9	72.6	23.7	36.9	60.8
	(F) PA Vert Center	39.3	2.7	34.9	78.0	27.3	42.3	67.3
	(G) AP–PA horizontal	40.9	10.3	27.8	71.2	22.6	37.9	98.7
Cerebellum	(A) Supratentorial	20.5	0.0	0.3	35.1	14.7	21.5	36.5
	(B) PA horizontal	69.8	40.1	62.9	82.2	35.9	62.9	159.4
	(C) PA horiz RT	68.1	38.7	61.8	82.0	35.0	61.4	155.8
	(D) PA horiz LT	68.1	38.4	62.3	82.3	35.5	61.9	155.4
	(E) PA VRT SUP	38.2	8.0	34.9	65.9	18.3	36.7	79.5
	(F) PA Vert Center	51.3	25.2	48.2	75.9	26.0	48.1	114.4
	(G) AP–PA horizontal	69.1	39.5	62.6	82.3	35.7	62.4	158.0

ROI	Array configuration	Current density PQM						
		CD_AUC_	V_CD15_	V_CD10_	V_CD5_	CD_75%_	CD_50%_	CD_5%_
	Units	(A/m^2^)	(%)			(A/m^2^)		
GTV	(A) Supratentorial	7.7	4.8	20.4	70.5	3.4	8.3	14.9
	(B) PA horizontal	11.6	25.5	49.8	82.5	6.3	10.0	26.5
	(C) PA horiz RT	11.3	24.3	47.9	81.9	6.2	9.6	25.7
	(D) PA horiz LT	11.4	24.5	48.8	82.4	6.2	9.8	25.6
	(E) PA VRT SUP	9.3	17.2	47.1	70.1	3.3	9.6	17.8
	(F) PA Vert Center	10.5	23.1	55.7	79.3	5.5	11.0	18.7
	(G) AP–PA horizontal	11.7	25.3	49.8	82.7	6.5	9.9	26.3
Cerebellum	(A) Supratentorial	3.4	0.0	0.1	16.4	2.0	3.5	5.9
	(B) PA horizontal	11.4	28.1	48.9	73.5	4.7	9.8	26.0
	(C) PA horiz RT	11.1	26.6	47.5	73.2	4.6	9.5	25.6
	(D) PA horiz LT	11.1	26.4	48.2	73.3	4.7	9.7	25.5
	(E) PA VRT SUP	6.5	5.5	20.2	54.0	2.5	5.7	15.3
	(F) PA Vert Center	8.4	14.0	36.4	64.6	3.6	7.5	17.8
	(G) AP–PA horizontal	11.3	27.5	48.5	73.4	4.7	9.7	25.7

**Fig. 3:**
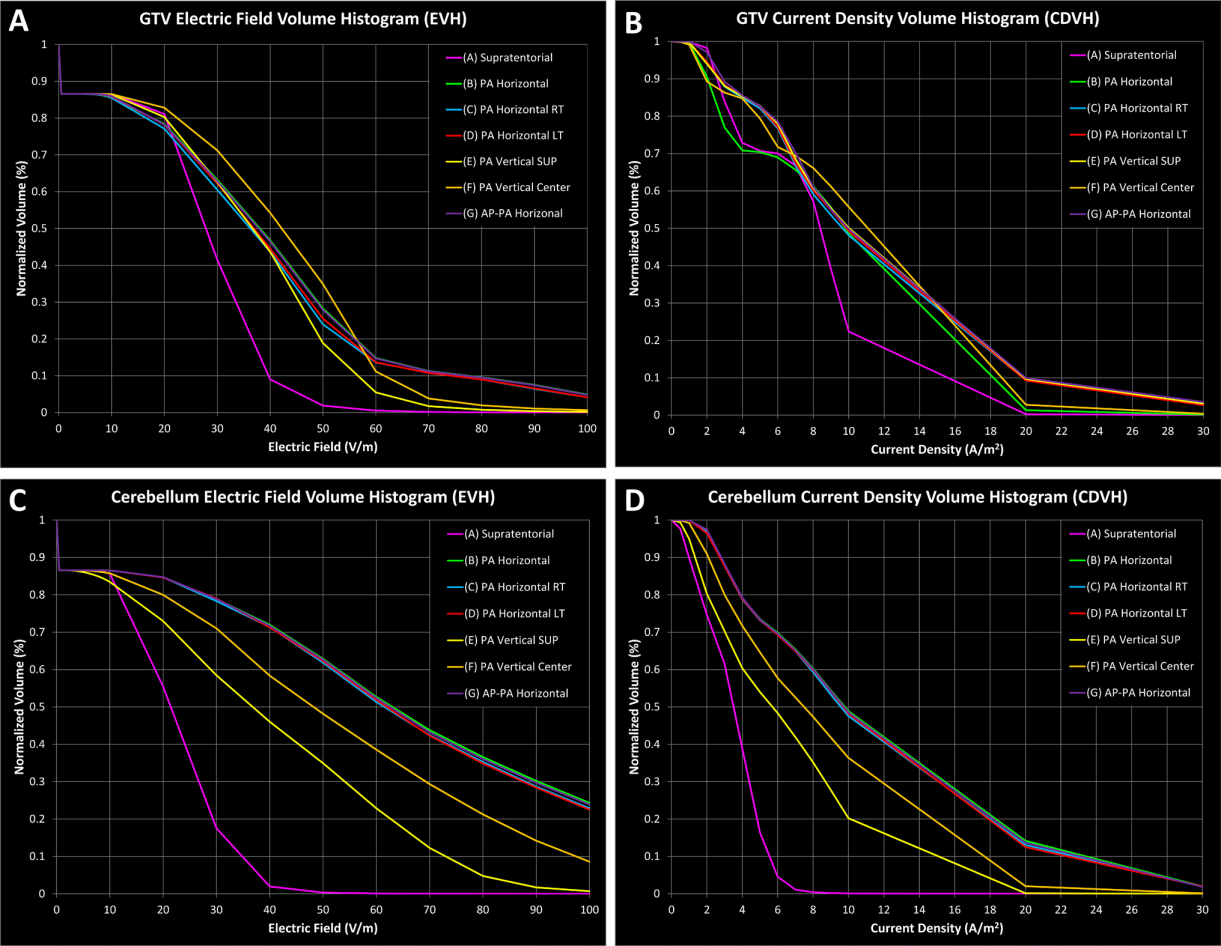
EVH and SARVH generated from the various array configurations for GTV and cerebellum. **a** EVH of the GTV for the various array configurations. **b** CDVH of the GTV for the various array configurations. **c** EVH of the cerebellum for the various array configurations. **d** CDVH of the cerebellum for the various array configurations

Out of all of the alternative array configurations in this study, the configuration with the least electric field coverage to the GTV when compared to the supratentorial array configuration, was the PA_Vertical-Superior_ configuration (Fig. 1e). The overall field intensity of this alternative configuration yielded an E_AUC_ of 34.4 versus 26.4 V/m for the supratentorial array configuration (Fig. 3a and Table 1), or a 30.1% increase. The PA_Vertical-Superior_ array configuration also produced a median electric field intensity E_50%_ of 36.9 vs 28.0 V/m (Fig. 3a and Table 1), or a 31.9% increase, and a CD_AUC_ of 9.3 versus 7.7 A/m^2^ (Fig. 3b and Table 1), or a 20.3% increase. Interestingly, the median CD_50%_ of the GTV for both PA_Vertical-Superior_ and PA_Horizontal-RT_ array configurations was similar, 9.6 versus 8.3 A/m^2^ (Fig. 3b and Table 1), or a 15.9% increase in median current density.

In contrast, of the 6 alternative array configurations modeled, the arrangement that provided the largest increase in field coverage at the GTV was observed when the PA array was positioned centrally along the patient’s posterior neck and horizontally parallel, along the longer axis of the array, to the axial plane of the head, or the PA_Horizontal_ configuration (Fig. 1b). This configuration yielded an E_AUC_ of 41.0 versus an E_AUC_ of 26.4 V/m in the supratentorial configuration (Fig. 3a and Table 1), or a 55.2% increase in overall electric field coverage. Similarly, this configuration produced a 36.3% increase in E_50%_, 38.1 versus 28.0 V/m (Fig. 3a and Table 1), respectively. Interestingly, the highest median electric field intensity within the GTV (42.3 V/m) was observed in the PA_Vertical-Center_ configuration. Likewise, the PA_Horizontal_ configuration had a 50.3% increase in overall current density CD_AUC_ when compared to the supratentorial configuration, 11.6 versus 7.7 A/m^2^ (Fig. 3b and Table 1) respectively. The CD_50%_ for the PA_Horizontal_ configuration was 10.0 A/m^2^ while the CD_50%_ for the supratentorial configuration was 8.3 A/m^2^ (Fig. 3b and Table 1), or a 20.5% increase in median current density coverage to the GTV. Interestingly, the PA_Vertical-Center_ configuration also produced the highest median current density of 11.0 A/m^2^ in the GTV (Fig. 3b and Table 1).

The AP array is complementary to the PA_Horizontal_ array and therefore the AP array position may alter the electric field and current density. To investigate this, we rotated the AP array by 90° in the AP-PA_Horizontal_ array configuration and solved for various electric field and current density parameters in the model. Both AP-PA_Horizontal_ and PA_Horizontal_ configurations had comparable E_AUC_ metrics, 40.9 versus 41.0 V/m respectively or a difference of 0.4%, and E_50%_ metrics, 37.9 versus 38.1 V/m respectively or a difference of 0.5% (Fig. 3a and Table 1). Similarly, both configurations had comparable CD_AUC_ metrics, 11.7 versus 11.6 A/m^2^ respectively or a difference of 0.4%, and CD_50%_ metrics, 9.9 versus 10.0 A/m^2^ respectively or a difference of 0.1% (Fig. 3b and Table 1). Other alternative array configurations yielded comparable electric field and current density coverage. Collectively, the computer simulation data indicate that any one of the posterior fossa configuration provided improved electric field delivery to our patient’s cerebellar glioblastoma compared to the standard supratentorial configuration.

### Hotspots are proportional to TTFields coverage of the GTV in the posterior fossa

Hotspots are generally regions or a percentage volume of a particular ROI that receives a greater quantity than that prescribed. Since TTFields do not currently have a clinically relevant threshold dose, 5% of the ROI receiving the highest TTFields intensity was chosen as the percentage volume representing a hotspot within that ROI and this is denoted as the E_5%_. The average electric field hotspot within the GTV was 44.1 V/m using the supratentorial array configuration while the average for the alternative array configurations was 86.2 V/m, or an average increase of 95.6%.

The alternative array configuration with the lowest hotspot intensity within the GTV was PA_Vertical-Superior_, with E_5%_ of 60.8 V/m compared to 44.1 V/m from the supratentorial configuration (Fig. 3a and Table 1), respectively, or a 38.1% increase. In contrast, the alternative array configuration with the highest hotspot intensity within the GTV was the PA_Horizontal_ configuration with E_5%_ of 99.1 V/m compared to 44.1 V/m from the supratentorial configuration (Fig. 3a and Table 1), respectively, or a 125.0% increase.

### Array positioning significantly alters TTFields coverage within the cerebellum

Although the main ROI for TTFields therapy is the GTV, it is also informative to observe and compare differences in TTFields distribution within adjacent normal tissue structures such as the cerebellum in this particular study. With an E_AUC_ of 20.5 V/m in the supratentorial array configuration, the average E_AUC_ for the alternative array configurations in aggregate was 60.8 V/m (Fig. 3c and Table 1), or a 197.1% increase. The median electric field within the cerebellum for the supratentorial configuration yielded an E_50%_ of 21.5 V/m compared to an E_50%_ of 36.7 V/m, or 70.7% increase, for the PA_Vertical-Superior_ and an E_50%_ of 62.9 V/m, or 192.6% increase, for the PA_Horizontal_ configuration, which were the alternative array configurations with the smallest and largest field intensity change, respectively (Fig. 3c and Table 1). Similarly, the supratentorial array configuration produced a CD_AUC_ of 3.4 A/m^2^ while the average CD_AUC_ in the alternative array configurations was 10.0 A/m^2^ (Fig. 3d and Table 1), or a 192.6% increase on average. The alternative array configuration with the smallest median current density was the PA_Vertical-Superior_ with a CD_50%_ of 5.7 A/m^2^ while the PA_Horizontal_ array configuration yielded the largest median current density of CD_50%_ of 9.8 A/m^2^, compared to a CD_50%_ of 3.5 A/m^2^ for the supratentorial array configuration (Fig. 3d and Table 1), or a 61.4% increase versus a 176.1% increase, respectively.

Electric field hotspots in the cerebellum measured by the E_5%_ was 36.5 V/m for the supratentorial array configuration while the E_5%_ for PA_Vertical-Superior_ was 79.5 V/m, or a 117.6% increase, and the E_5%_ for PA_Horizontal_ was 159.4 V/m, or a 336.3% increase (Fig. 3c and Table 1).

Similarly, current density hotspots within the cerebellum measured by the CD_5%_ was 5.9 A/m^2^ for the supratentorial array configuration while the CD_5%_ was 15.3 A/m^2^, or a 157.3% increase, and 26.0 A/m^2^, or a 338.2% increase, for the PA_Vertical-Superior_ and the PA_Horizontal_ posterior fossa array configurations, respectively (Fig. 3d and Table 1).

## Discussion

TTFields therapy is an accepted treatment modality for patients with glioblastoma and a better understanding is needed on array positioning that can affect TTFields coverage at the tumor target. This is particularly important for glioblastomas located within the posterior fossa and, to our knowledge, we are first to show that the standard array configuration does not provide adequate electric field coverage for the GTV located in this region. However, the alternative array configurations for the posterior fossa provided an average of 48.0% more coverage to the GTV as measured by the E_AUC_. In addition, hotspots defined by the E_5%_ had an average increase as much as 95.6%. Although only 4% of adult gliomas are located in the posterior fossa and the biology of these tumors is probably different from those located in the supratentorial brain [10], posterior fossa glioblastomas may still benefit from TTFields. This is because the mechanism of TTFields’ anti-tumor effect applies to any dividing tumor cells where large proteins with high dipole moments are required for cytokinesis and segregation of sister chromatids during metaphase and anaphase in mitosis [2, 11]. With specific placement of the arrays as part of treatment planning, TTFields can potentially become a precision-guided anti-tumor therapy targeting dividing glioblastoma cells at a pre-specified location in the brain. Therefore, determining the electric field coverage of the glioblastoma within the intracranial space is highly relevant to patient care.

Array positioning has been shown to affect the electric field strength at various intracranial structures and particularly at the GTV [7, 12]. To quantify this effect, we used a set of PQM parameters derived from the EVH and CDVH of different models. PQM has been used to evaluate radiation treatment plans to ensure adequate coverage to the target(s) while minimizing doses to the surrounding normal tissue [13, 14]. For our application in TTFields, relevant parameters include E_AUC_ and CDVH_AUC_ for an aggregate measure of electric field and current density coverage at the GTV, as well as measurements of E_5%_ and CDVH_5%_ at hotspots within the GTV, respectively. Indeed, using this method, we were able to compare quantitatively the strength of the electric fields and current densities across a number of array configurations.

A similar analysis consisting of computing TTFields intensity and power distribution for supratentorial glioblastomas was performed utilizing MRI data from subjects participated in the EF-14 randomized clinical trial [3]. Using a parameter of local minimum dose density at the GTV, which is defined as the product of TTFields intensity, tissue-specific conductivities and patient compliance, a correlation was found between dose density and survival [15]. However, only 340 (73%) of the entire TTFields-treated population (n = 466) had MRI qualities acceptable for analysis and the outcome of this analysis remains to be confirmed in a validation cohort.

Our finite element analysis revealed that TTFields coverage of the cerebellar glioblastoma was greatly improved with any of the alternative array configurations for the posterior fossa when compared to the standard supratentorial configuration. This is most likely due to the increased proximity of the PA array to the tumor, which is located at the dorsal region of the posterior fossa. Of the 6 alternative configurations, the PA_Horizontal_ array configuration provided the most extensive coverage to the GTV. This benefit is most likely attributed to coverage of the entire GTV with increased electric fields provided by the PA array. Specifically, the PA_horizontal_ configuration, in comparison to the PA_Vertical-Superior_ and PA_Vertical-Center_ configurations, decreases the distance between the PA and the two lateral arrays, and therefore increases the electric field intensity within the posterior fossa. This increase in field strength has been observed by other studies performing similar TTFields modeling [7, 16]. A probable and additional attribution to the increased coverage could be due to the slight angular rotation and posterior shift of the right and left lateral arrays, providing a higher current density throughout the posterior portion of the brain and thereby increasing electric field coverage to the GTV.

By applying the fields to the head using the PA_Horizontal_ configuration, our computer modeling revealed a qualitative increase in the electric field penetration at the GTV, compared to the PA_Vertical-Superior_ and PA_Vertical-Center_ configurations, when the PA array was rotated so that the long axis of the array was parallel to the coronal plane. However, this array position also increased the field intensity within the vertebral bodies and muscles posteriorly in the neck. As shown in Fig. 2b–d, the three horizontal PA configurations produced higher field intensities at the odontoid of the C2 vertebral body when compared to the PA_Vertical-Superior_ and PA_Vertical-Center_ configurations, and even more so when compared to the supratentorial configuration. In addition, a qualitative assessment of models applying the PA_Horizontal_, PA_Horizontal-RT_ and PA_Horizontal-LT_ configurations produced an increase in field intensity within the scalp region inferiorly but a decrease in field intensity superiorly. When shifted laterally left or right by 2 cm from the PA_Horizontal_ configuration, only marginal and probably non-clinically relevant differences in field coverage of the GTV were observed. Additionally, by rotating the AP array by 90° in the AP-PA_Horizontal_ configuration, only marginal differences were observed in fields coverage quantified by the various PQM metrics. This suggests that rotating the AP array, potentially provides another means of applying roughly the same field intensity as the PA_Horizontal_ configuration for tumors in the posterior fossa (Fig. 3).

An increase in the electric field intensity was also observed at the genu of the corpus callosum and the anterior one-third of the body of this structure in the alternative array configurations. This is likely due to the fact that the most inferior margin of the fields extends well beyond the posterior commissure line and thus TTFields cover a greater area of the corpus callosum. However, in the supratentorial array configuration, the margin of the field only tangentially skims the posterior commissure line.

A limitation of our finite element modeling is a lack of experimentally measured electric field data for both supratentorial and alternative array configurations in the posterior fossa. This will require a clinical trial in which the electric field intensity is measured in the patient while TTFields are being applied to the scalp. Furthermore, the exact conductivity and permittivity values for glioblastoma are unknown. However, our prior sensitivity analysis has shown that the electric field strength from modeling is primarily influenced by tissue conductivity rather than permittivity [12] and to obtain tumor-specific conductivity value will require experimental measurements as well.

In summary, this is the first finite element modeling of a cerebellar glioblastoma and we showed that the alternative array configuration for the posterior fossa offers an improved TTFields coverage to the tumor compared to the conventional supratentorial array configuration. This increased electric field coverage is due to shifting of the PA array to the lower occipital and upper cervical regions while the right and left lateral arrays are moved backward. Benefit of this array positioning will require clinical validation.
